# Protocol for precision cutting and short-term culture of lymph node tissue slices for modeling of innate immune responses *ex vivo*

**DOI:** 10.1016/j.xpro.2026.104421

**Published:** 2026-03-11

**Authors:** Joannah R. Fergusson, Mark Coles, Calliope A. Dendrou, Anita Milicic

**Affiliations:** 1 Kennedy Institute of Rheumatology, University of Oxford, Roosevelt Drive, Oxford OX3 7FY, UK; 2 Jenner Institute, University of Oxford, Old Road Campus Research Building, Roosevelt Drive, Oxford OX3 7DQ, UK

**Keywords:** cell culture, cell-based assays, immunology

## Abstract

Secondary lymphoid organs are highly organized for the initiation of immune responses. Here, we present a protocol that enables this microanatomical structure to be preserved during short-term culture, allowing *ex vivo* modeling of human innate immune responses. We describe the steps for precision cutting of human lymph nodes into live tissue cross-sections that can be cultured for up to 20 h with immune stimuli and analyzed by multiple readouts.

For complete details on the use and execution of this protocol, please refer to Fergusson et al.^1^

## Highlights

• Steps for precision cutting of live human lymph node tissue slices
• Instructions for short-term culture of tissue slices with innate immune stimuli
• Guidance for downstream analyses to determine mechanisms of action of immune stimuli

## Abstract (editor highlights)

Publisher’s note: Undertaking any experimental protocol requires adherence to local institutional guidelines for laboratory safety and ethics.

## Graphical abstract

**Figure:**
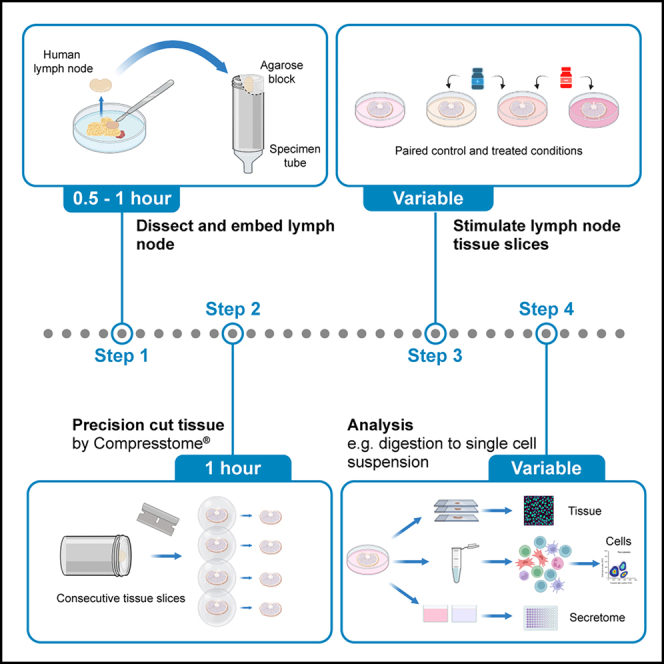

## Before you begin

**Figure 1:**
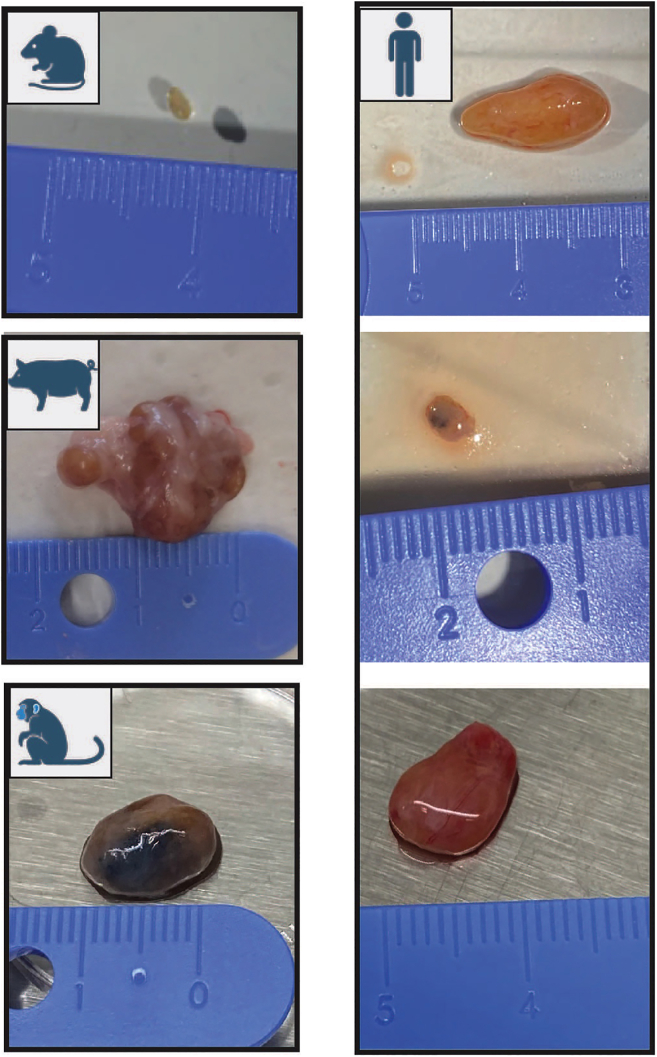
Variation in size and shape of lymph nodes Left displays an example lymph node from mouse (inguinal), pig (mesenteric) and primate (axillary). Right shows cystic lymph nodes from three separate human donors.

This protocol describes the specific steps for precision cutting and short-term culture of human lymph node tissue slices and analysis of their responses to immune stimuli such as a vaccine adjuvant. This protocol retains viability of lymph node tissue for up to 20 h enabling the study of innate immune responses. Precision cutting is performed using a Compresstome®. Human lymph nodes used in this study were obtained as a by-product of routine cholecystectomy. We have also used this protocol with lymph nodes from other species (mouse, pig, primate), which vary in size, shape and tissue density. Human cystic lymph nodes, resected from gallbladder-surrounding tissue following cholecystectomy, also display variation between patients (Figure 1). In such cases, small adjustments may be made to the protocol which are detailed in the notes accompanying the relevant steps.

The Compresstome® is of a small enough footprint to fit within a tissue culture hood to maintain sterility. However, we have performed lymph node tissue dissection (step 1), embedding (step 6) and precision cutting (steps 9–23) on the benchtop without microbial contamination of subsequent tissue slice cell cultures. The risk of contamination is reduced by keeping all media components sterile by use of a tissue culture hood, specifically for culture media preparation and tissue slice collection (step 24) and culture (steps 25–27), and by the addition of antibiotics to PBS used within the Compresstome® buffer tray during the cutting (step 11).

### Prepare equipment and reagents

**Timing: 30 min**

1. Prepare solution of 2.5% Agarose in PBS (do not heat to dissolve until tissue is ready).

***Note:*** The agarose consistency should match the tissue density as closely as possible, and a percentage of 2.5% agarose has proven optimal for the cutting of lymph nodes across tested species.

***Optional:*** The use of low gelling temperature (temp.) agarose allows for the positioning of lymph nodes in the agarose before the gel sets, and has been used successfully with mouse lymph nodes. However, low gelling temp. agarose has proven too soft for the cutting of lymph nodes from larger mammals, where the use of standard agarose provides better embedding for cutting.

2. Prepare PBS + 1% Penicillin/Streptomycin (Pen/Strep) for the Compresstome® buffer tray, chill at 4°C.
3. Place the Compresstome® chilling block in a −20°C freezer for at least 1 h.
4. Prepare a 24 well plate containing one set of the following solutions (a-c), at 19°C–22°C:
  a. 1 mL of 0.01% Digitonin in PBS.
  b. 1 mL of PBS.
  c. 1 mL of PBS (fresh well).
5. Prepare culture media, and aliquot 1 mL into wells of two 24 well plates at 19°C–22°C: one plate for tissue slice collection and one plate for resting the cut tissue slices. A further plate may be prepared containing the immune stimulant of choice for tissue slice culture (step 26). Keep culture media sterile.

### Innovation

This protocol enables a viable short-term culture of human lymph nodes through precision cutting of live tissue cross-sections. We present a protocol specifically optimised for the slicing of human lymph nodes, which are larger and more elastic and hence significantly more difficult to cut than mouse lymph nodes slices.^2^^,^^3^ Given the relative scarcity of human tissue, the presentation of an optimized protocol enables researchers to directly use such tissue for functional analyses of human innate immune function, or for further culture techniques.^4^ Expanding on 2D cell culture and organoid approaches, tissue slices preserve the 3D lymphoid microarchitecture, allowing physiologically relevant insights into immune perturbation. By using cross-sections of the full organ, precision-cut slices also enable broad representation of lymph node substructures in a standardised manner. Importantly, the generation of multiple tissue slices from each donor allows paired analyses of untreated and treated conditions, important in the context of donor-to-donor variation. Guidance for downstream analysis is also provided – including immunohistochemistry, secretome analysis and preparation of single-cell suspension for flow cytometry or transcriptomics – presenting a comprehensive model for mechanistic studies of human lymphoid tissue.

### Institutional permissions

Human lymph nodes were obtained from adult patients undergoing routine cholecystectomy for symptomatic gallstones, a non-inflammatory condition. Lymph nodes were resected from the tissue surrounding the excised gallbladder and processed within 2 h of surgery. Use of human tissue requires ethical approval from relevant institutions, and processing of tissue and patient details according to ethical guidelines and local laws. In our work, written informed consent was obtained from each patient, and samples were collected under REC 21/YH/0206 as approved by Yorkshire and The Humber - Sheffield Research Ethics Committee, UK.

## Key resources table

**Table:** 

REAGENT or RESOURCE	SOURCE	IDENTIFIER
**Antibodies**		

Human IL-12 p70 Antibody (5 μg/mL for blocking)	R&D Systems	Cat #: MAB219; RRID: AB_2123616; Clone #: 24910
Human IL-18/IL-1F4 Antibody (5 μg/mL for blocking)	R&D Systems	Cat #: D044–3; RRID: AB_356964; Clone #: 125–2H
PE anti-human CD235a (Glycophorin A) Antibody (dilution 1:100)	BioLegend	Cat #: 349105; RRID: AB_10641707; Clone: HI264

**Biological samples**		

Human Lymph Nodes	Cholecystectomy by-product	REC: 21/YH/0206

**Chemicals, peptides, and recombinant proteins**		

RPMI 1640 Medium	ThermoFisher Scientific (Gibco)	Cat#: 21875–034
Dispase II, powder	ThermoFisher Scientific	Cat#: 17105–041
Collagenase P	Merck	Cat#: 11213857001
Deoxyribonuclease I from bovine pancreas	Sigma Aldrich	Cat#: D5025–150KU
Fetal Bovine Serum (FBS), Value, One Shot™ format	ThermoFisher Scientific (Gibco)	Cat#: A5209401
UltraPure 0.5 M EDTA, pH 8.0	ThermoFisher Scientific (Invitrogen)	Cat#: 15575020
Digitonin (5%)	ThermoFisher	Cat#: BN2006
UltraPure Agarose	ThermoFisher Scientific (Invitrogen)	Cat#: 16500500
Penicillin-Streptomycin (10,000 U/mL)	ThermoFisher Scientific (Gibco)	Cat #: 15140122
PBS, pH 7.4	ThermoFisher Scientific (Gibco)	Cat #: 10010015
Sodium Pyruvate	Agilent	Cat #: 103578–100
MEM Non-Essential Amino Acids (100×)	ThermoFisher Scientific (Gibco)	Cat #: 11140035
HEPES (1M)	ThermoFisher Scientific (Gibco)	Cat #: 15630–056
Adjuvant LMQ	Vaccine Formulation Initiative	O’Donnell et al.^5^
eBioscience Cell Stimulation Cocktail (500×)	ThermoFisher Scientific (Invitrogen)	Cat #: 00-4970-93
TLR4 inhibitor, TAK242	Merck	Cat #: 614316
MCC950 (CP-456773 sodium salt)	Sigma Aldrich	Cat #: PZ0280
eBioscience Protein Transport Inhibitor Cocktail (500×)	ThermoFisher Scientific (Invitrogen)	Cat #: 00-4980-93
Formalin solution, neutral buffered, 10%	Sigma-Aldrich	Cat #: HT5011

**Critical commercial assays**		

LEGENDPlex™ Human Inflammation Panel 1 (13-plex) with V-bottom plate	BioLegend	Cat #: 740809

**Other**		

Compresstome® VF-510-0Z	Precisionary Instruments	Model: VF-510-0Z
Stainless Steel Blades	Precisionary Instruments	SKU: VF-BL-VM-SSB-CANAL
Brushes for Tissue Handling	Precisionary Instruments	SKU: VF-VM-PB-CANAL
Dissection tools e.g., forceps, scissors	InterFocus	e.g., 11200-14, 15040-11
Stereo Microscope e.g., M80 Series	Leica	N/A
Corning® tissue-culture treated culture dishes	Merck	Cat #: CLS430165
Digital thermometer	Digi-Sense	Product code: 86460-01
Eppendorf ThermoMixer® Compact	Sigma Aldrich	Product: T1317
Falcon™ Cell Strainers, 100 μm sterile	Fisher Scientific	Cat #: 10282631
Fisherbrand™ Sterile Syringes, 1 mL	Fisher Scientific	Cat #: 15889142
Q Path® Biopsy pads	VWR	Cat #: 720-2254
Biopsy Cassettes	Fisher Scientific	Cat #: 16333986

***Alternatives:*** A reusable ceramic blade (Precisionary Instruments, SKU: VF-BL-VM-CB-CANAL) may be used in place of stainless steel blades with the Compresstome VF-510-0Z.

## Materials and equipment

**Table:** 2.5% Agarose

Reagent	Final concentration	Amount
PBS	N/A	40 mL
Agarose	2.5%	1 g

Can be stored at 19°C–22°C and reheated up to 3 times.

**Table:** Culture Media

Reagent	Final concentration
RPMI 1640	N/A
FBS	10%
Penicillin/Streptomycin	100 U/mL
Sodium Pyruvate	1 mM
MEM Non-Essential Amino Acids (100×)	1×
HEPES	20 mM

Store at 4°C for up to 1 month.

**Table:** Enzyme Mix – Make up 3 mL per tissue slice

Reagent	Final concentration
RPMI 1640	N/A
Dispase	0.8 mg/mL
Collagenase-P	0.2 mg/mL
DNase	0.1 mg/mL

Can be made in advance and stored at 4°C for up to 1 day.

**Table:** Cell Collection Buffer – Make up 3 mL per condition or tissue slice (depending on whether pooling of tissue slices is intended)

Reagent	Final concentration
PBS	N/A
FBS	2%
EDTA	2 mM

Store at 4°C for up to 1 month.

## Step-by-step method details

### Embed lymph nodes in agarose for precision cutting

**Timing: 30 min to 1 h**

This step prepares the tissue for precision cutting by embedding it in an agarose block.

**Figure 2:**
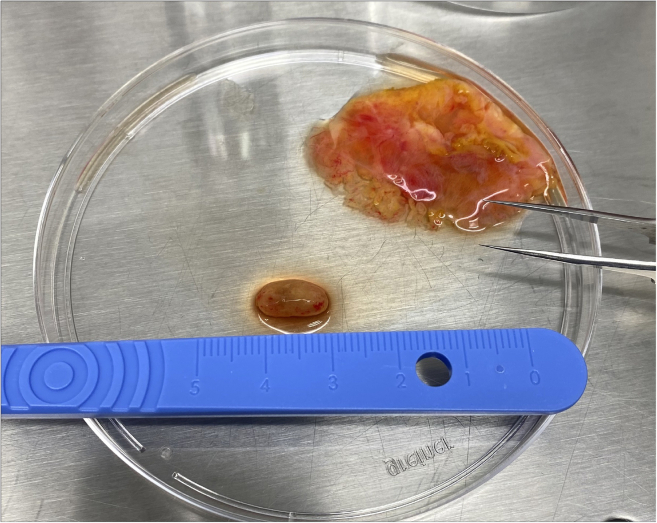
Dissection of human lymph node from surrounding connective and adipose tissue

1. Within 2 h of surgery, transfer the lymph node to a 35 mm dish and dissect it from the surrounding tissue, without piercing the lymph node (Figure 2).

***Note:*** Use of a dissection microscope, such as a Stereomicroscope, may aid in removing as much fat as possible.

2. Place the dissected lymph node in PBS in a 24 well plate and keep at 19°C–22°C.
3. Dissolve or melt the agarose solution in a microwave.

***Note:*** Agarose may be melted and reused up to 3 times, beyond this the evaporation levels alter the agarose concentration to the point that embedding becomes difficult. Evaporation can be limited by placing cling film over the neck of the container during heating, with one small hole punched through to allow escape of steam.

**Figure 3:**
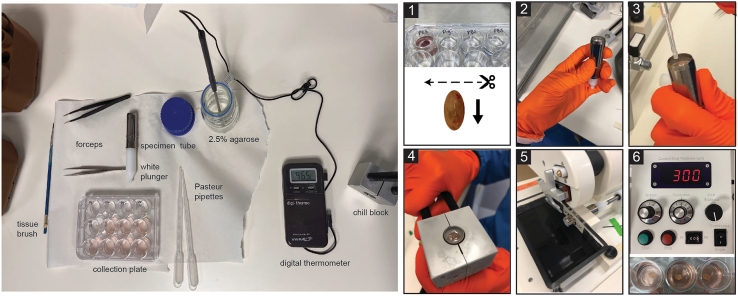
Overview of components required for tissue embedding (left) and the embedding and cutting procedure (1–6)

4. Leave the agarose to cool, monitoring the temperature with a digital thermometer.
5. Using forceps, consecutively transfer the lymph node between the pre-prepared wells containing the following solutions at 19°C–22°C (Figure 3-1) and for the indicated lengths of time:
  a. 0.01% Digitonin in PBS for 1–2 s.
  b. PBS for 7–10 s.
  c. PBS (fresh well) until ready to embed.

***Note:*** A digitonin wash acts to remove residual lipid droplets from the surface of the lymph node, which helps the embedding and tissue retention within the agarose during cutting, without deleterious effects on tissue viability, phenotype or responses to stimuli, as has been described with mouse lymph nodes following vaccination.^6^ However, the time in the digitonin detergent should be kept to a minimum.

**Figure 4:**
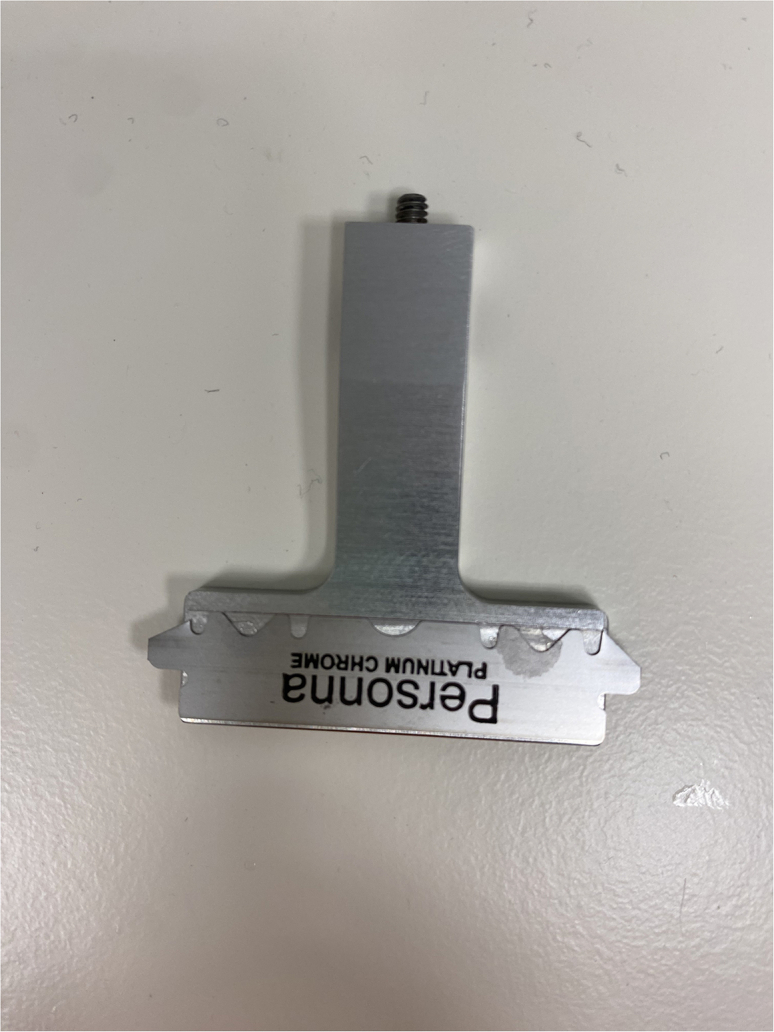
Correct loading position of a stainless-steel blade on the magnetic blade holder

6. Once the agarose solution has cooled to 45°C, measured with a thermometer, use a Pasteur pipette to fill the specimen tube and then embed the tissue:
  a. Draw the white plunger of the specimen tube downwards and hold in place to allow the tube to be filled with enough agarose to fully cover the specimen once it’s added (Figure 3-2).
    ***Note:*** Be careful not to introduce air bubbles, removing any by tapping the side, or suctioning out with a Pasteur pipette.
  b. Using forceps, take the lymph node out of the PBS and blot on tissue paper to remove residual liquid.
  c. Add the lymph node to the center of the agarose.
  d. Top up with agarose as needed (Figure 3-3).
    ***Note:*** Human lymph nodes vary in size and shape but are generally easiest to cut along the transverse plane, with the blade passing through the narrowest part of the tissue (Figure 3-1). Embed the lymph node in a direction which allows cutting in this orientation.
    **CRITICAL:** Once in the specimen tube, the agarose solution cools quickly, such that at the point of adding the tissue the agarose should be at ∼37°C, avoiding tissue heat shock. However, it is critical to work quickly and to add the tissue before the agarose sets. Topping up with extra agarose should also be performed quickly to prevent any separation of the block due to prior setting of the initial agarose.
7. Place the specimen tube in the chill block, being careful to hold the white plunger in place without movement.
8. Hold the specimen tube in the chill block until the agarose turns opaque and has set (Figure 3-4). Once set, place on ice until ready.

### Precision cutting of lymph nodes into tissue slices

**Timing: 1 h (per lymph node)**

This step generates precision cut tissue slices of desired thickness from the human lymph node. Variations in operation may occur depending on the model of Compresstome®, and may be referred to in the manufacturer’s protocol. The model used here is the VF-510-0Z.

**Figure 5:**
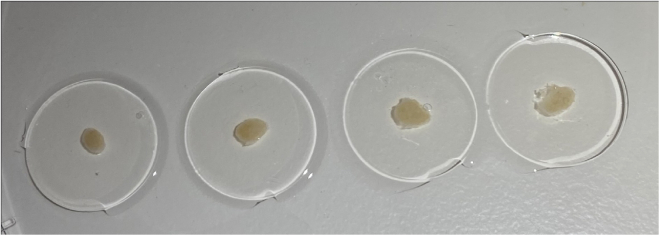
Consecutive live lymph node slices in agarose

**Figure 6:**
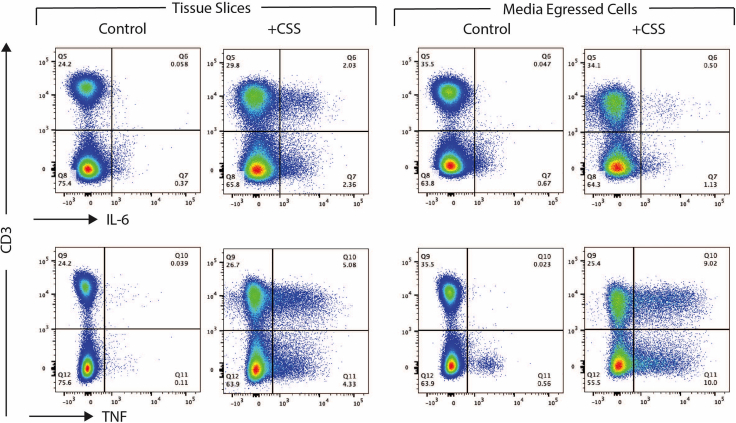
Flow cytometric analysis Control and Cell Stimulation Cocktail (CSS)-stimulated human lymph node tissue slices were analyzed after 20 h, with protein transport inhibitor cocktail added for the final 4 h of culture. Cells were collected from tissue slices by digestion (left; Tissue Slices) or from egressed cells by collecting the cell pellet following centrifugation of the culture supernatant (right; Media Egressed Cells). Cells were analyzed for intracellular expression of IL-6 or TNF.

9. Slide the Compresstome® control box back to create space to mount the specimen tube.
10. Place the specimen tube containing the embedded lymph node into the buffer tray of the Compresstome®, pushing it in fully by the metal tubing (rather than by the white plunger) until the stopper ring is flush with the buffer tray.
11. Fill the buffer tray with the chilled PBS + Pen/Strep, ensuring the specimen is fully submerged (Figure 3-5).
12. Move the control box back into place, and then, using the fast forward (FF) button, move the micrometer (tissue advancement plunger) forward until it is touching the back of the specimen tube base.
13. Using scissors, cut the double-edged stainless-steel blades horizontally through the middle into individual blades and trim the extra side tabs to allow the blade to be mounted straight.
14. Place one trimmed stainless-steel blade onto the magnetic blade holder, ensuring that it is flat, centered, and its top aligned against the top edge of the blade holder (Figure 4).
15. Attach the blade holder to the vibrating unit and lock into place with the provided Allen key.
  **CRITICAL:** Change the blade regularly, after each tissue specimen and halfway through larger specimens.

16. Trim the agarose down by sectioning on continuous mode until near the tip of the tissue. Thicker sections <500 μm of agarose may be cut at a speed of 6–8 and the frequency of oscillation at 6. (Methods video S1).
17. Continue cutting until full cross-sections of agarose are cut and the tissue is near the surface of the specimen tube, then stop continuous sectioning.
  ***Note:*** Slice thickness, cutting speed and oscillation frequencies are less important while trimming the agarose than when slicing through tissue, and may be performed at higher settings. The first few slices may not be full cross-sections of agarose as the surface of the agarose may not be level (Methods video S1).

18. Set the desired slice thickness for the embedded tissue, e.g., 300 μm.
  ***Note:*** Slice thicknesses of 300–400 μm generate consistent cross-sections from live human lymph nodes. Cutting of thinner slices is possible, but quality of consecutive slices is more variable i.e. may not produce complete cross-sections. Reduced viability of slices has been observed at thicknesses >500 μm.

19. Set the sectioning speed at 1–2 and the frequency of oscillation at 6 (Figure 3-6).
20. Proceed with cutting tissue slices on single cutting mode by pressing start (Methods video S2). Troubleshooting 1 and 2.

***Note:*** It is recommended that this is performed on single rather than continuous cutting mode, to allow collection of consecutive slices and manual trimming of any residual connective tissue with scissors, if needed.

***Note:*** Several slices of agarose only, without embedded tissue, may be generated initially until the blade reaches the tissue, and can be discarded. The first and last tissue slices will be of unknown thickness and are likely to consist largely of lymph node capsule. Subsequent tissue slices should be tissue cross-sections of the desired thickness.

21. Collect slices in agarose using the flat-headed forceps or a tissue brush, and place each slice into a separate well of a 24 well plate containing 1 mL culture media. (Methods video S3).
  **CRITICAL:** Number the slices as they are collected to keep track of original slice position within the lymph node.
  ***Note:*** The number of slices obtained varies with the size of the lymph node, with a human cystic lymph node yielding approximately 10–20 good quality tissue cross-sections. Some poorer quality slices, e.g. not full cross-sections or with tissue holes, may also be generated but should be discarded or used for optimisation.

22. Once the embedded tissue has been fully sliced, remove the cut tissue slices in agarose from the media using flat-headed forceps and/or a tissue brush and place onto a dry 35 mm petri dish (Figure 5).
23. Use straight forceps to cut through the agarose and a tissue brush to separate the tissue slice from the agarose.
24. Carefully transfer each slice into a new well containing 1 mL of culture media. Place each tissue slice into a separate well.
  ***Note:*** Removal of agarose may be aided by using a dissection microscope.
  ***Optional:*** Depending on the downstream application, tissue slices may be retained in agarose without impact on viability. Tissue slices may also have separated from the agarose during slicing (Troubleshooting 3), removing the need for this step.

**Figure 7:**
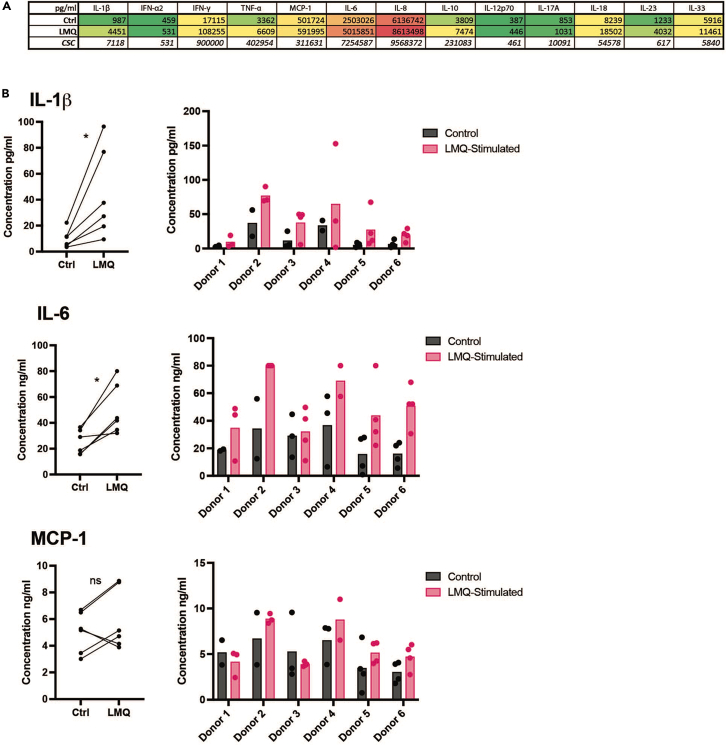
Cytokine concentrations detected in tissue slice culture supernatants after 20 h culture (A) Average cytokine levels in culture supernatants from control (Ctrl) and LMQ-stimulated human lymph node tissue slices (2–4 slices per condition; n=6 donors). A cell stimulation cocktail positive control (CSS) is shown for comparison. (B) Cytokine levels displayed as paired averages where each point is the average of 2–4 slices from each donor, assessed by Wilcoxon matched-pairs signed rank test, ∗p<0.05 (left), or as individual replicate values per slice and per donor (right).

**Figure 8:**
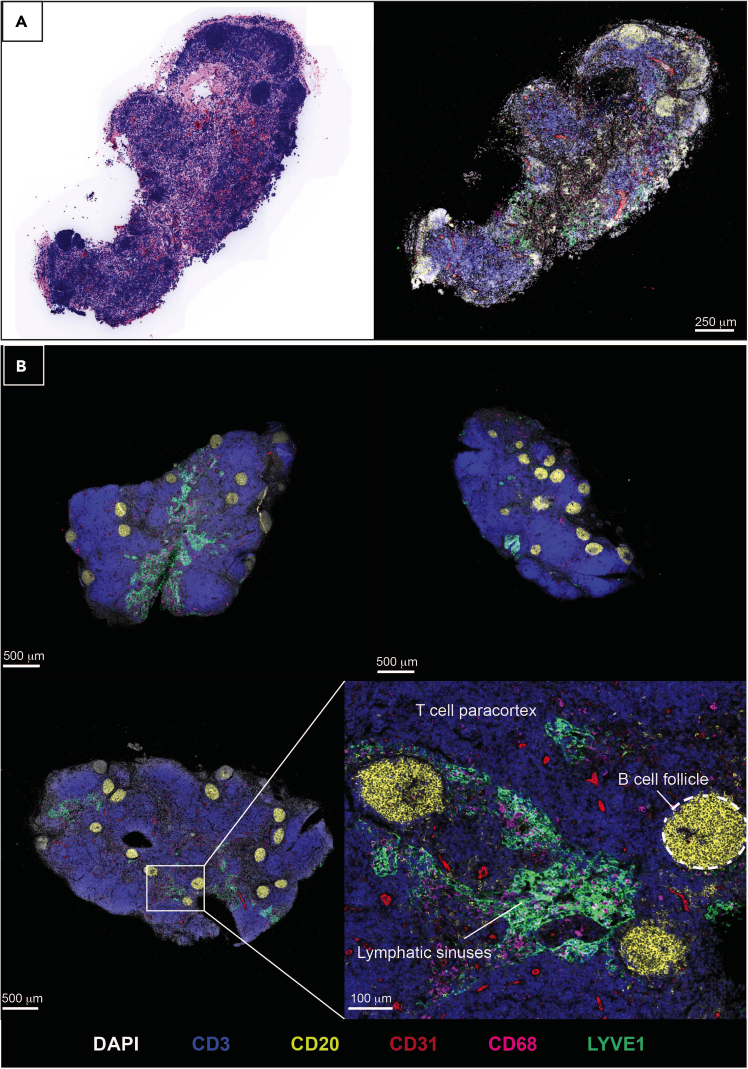
Human lymph nodes slices after culture stained by multiplexed immunohistochemistry Scale bars and markers are indicated. (A) A virtual hematoxylin & eosin stain and corresponding multiplexed stained image of the same lymph node slice. (B) Three lymph node slices from the same donor and inset zoomed-in view of maintained microarchitectural features, as labelled.

**Figure 9:**
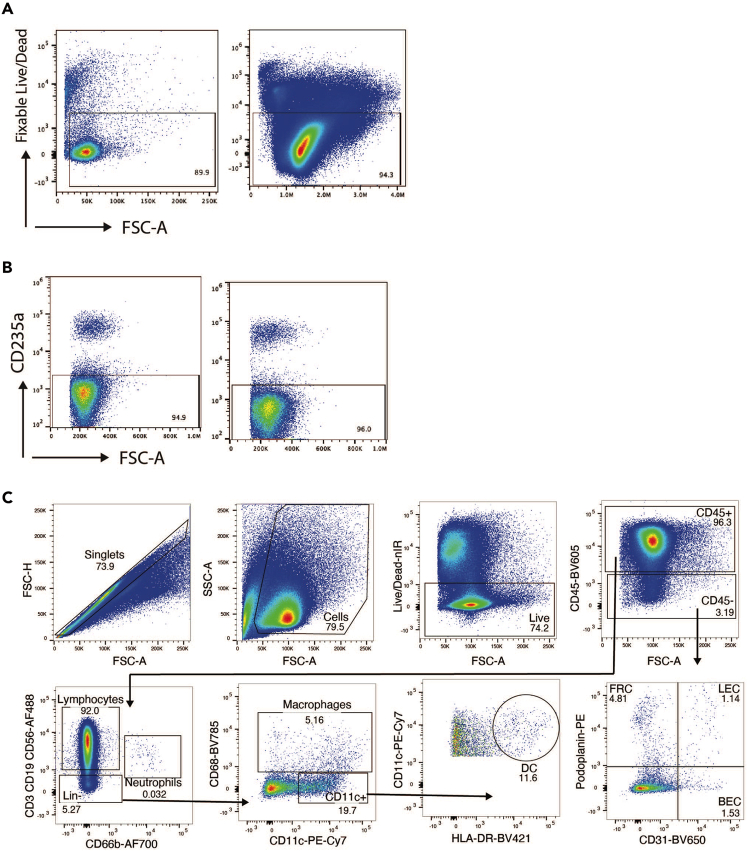
Flow cytometric analysis of single-cell suspension from digested human tissue slices, following 20 h culture (A) Live/Dead staining of human lymph node slices from two donors, gated on singlets only. Percentages of live cells are indicated. (B) CD235a staining to identify red blood cells in digested lymph node tissue slices. (C) Representative gating strategy to identify leukocytes and non-leukocytes in human lymph node tissue slices. Lin, lineage, DC, dendritic cell; FRC, fibroblastic reticular cell; LEC, lymphatic endothelial cell; BEC, blood endothelial cell.

### Stimulate lymph node slices

**Timing: 1.5–2.5 h**

25. Place the plate containing the tissue slices in a CO_2_ incubator at 37°C to rest for 1 h, to allow recovery of tissue and egress of dead cells and debris.
26. After the resting period, carefully transfer tissue slices, using a tissue brush and in a tissue culture hood, into 1 mL of fresh culture media containing the immune stimulant of choice. For example:
  a. use a dose of 12.5 μL of adjuvant LMQ which contains 2.5 μg of QS-21 saponin and 1 μg of TLR4-agonist 3D6AP,^1^ or
  b. 2 μL (1×) cell stimulation cocktail.
    ***Optional:*** For pre-treatment with molecular inhibitors or blocking antibodies, incubate slices in a CO_2_ incubator at 37°C in media containing the inhibitor/antibody for 1 h before directly adding the immune stimulant to the well, thereby keeping the inhibitor/antibody in culture for the duration. For instance, 1 μM MCC950 and/or 5 μM TAK242 inhibit production of IL-1β, while 5 μg/mL of anti-IL-18 antibody and 5 μg/mL of anti-IL-12p70 antibody inhibit IFNγ secretion, by human lymph node slices.
    **CRITICAL:** Distribute sequential tissue slices between conditions, i.e. slice 1 = control, slice 2 = treatment, slice 3 = control, slice 4 = treatment etc., to ensure fair representation of tissue structures and cell types for each condition. Ensure multiple slices (between 2 and 7 slices, depending on the total obtained) per treatment groups.
    ***Optional:*** For assessment of cytokine production by intracellular cytokine staining, 2 μL (1×) protein transport inhibitor may be added for the final 4 h of culture (Figure 6) or for the duration. However, addition of protein transport inhibitors will limit the secretion of mediators into the culture supernatant for analysis by ELISA or LEGENDPlex (Step 28a).

27. Incubate the plate in a CO_2_ incubator at 37°C for 20 h, or for the desired length of time. Incubation times should be optimized for each immune stimulant. Troubleshooting 4.

### Analysis of lymph node slice cultures

28. At the end of the culture period, slices and supernatant may be collected for analysis. For example:
  a. Collect culture supernatants in an Eppendorf. Centrifuge at 400 × *g* to pellet tissue-egressed cells and transfer the cell-free supernatant to a new Eppendorf for storage at −80°C. This supernatant may be analyzed for cytokine release by, for example, ELISA or LEGENDPlex. Untreated tissue slices provide baseline measurements of cytokine release, and culture media alone may be used as a blank control.
    ***Optional:*** The remaining cell pellet may be analyzed as tissue-egressed cells, for example by flow cytometry (Figure 6).
    **CRITICAL:** Given the potential for slice-to-slice variation in the response to stimuli, it is critical to take the average of replicates for each condition (as many as possible depending on number of slices generated, with at least two per condition). Mean responses from treated slices should then be compared to paired mean values of the control conditions (Figure 7).
  b. Fix tissue slices for 1 h at 19°C–22°C in at least 1 mL of 10% formalin solution and embed in paraffin for further sectioning and immunohistochemical staining, for example by standardized protocols, e.g.,^7^^,^^8^ (Figure 8). Troubleshooting 5.
    **Pause point:** Once fixed and paraffin embedded, tissue slices can be stored as blocks for years before analysis.
  c. Digest tissue slices into a single cell suspension for flow cytometric (Figures 6 and 9) or transcriptomic analysis, as described below.

### Digest lymph node slices into a single-cell suspension

**Timing: 2–3 h**

29. Prepare 3 mL of digestion mix per tissue slice or treatment group, and pre-warm to 37°C.
30. Prepare cell collection buffer, chill at 4°C.
31. Transfer the tissue slice into an Eppendorf containing 1 mL of pre-warmed digestion mix.
  ***Note:*** Tissue slices may be pooled into treatment groups at this point or each slice kept separate, according to downstream analyses.

32. Use scissors to cut the tissue slices into smaller pieces, as much as possible.
33. Incubate at 37°C with mixing (mixing speed 400 rpm) using an Eppendorf ThermoMixer or similar, for 15–20 min.
  ***Optional:*** If a heated mixer is not available, place in an incubator or water bath and mix by inversion every 5 min.

34. Mix the suspension using a Pasteur pipette.
35. Allow the tissue pieces to float to the bottom.
36. Using a Pasteur pipette collect as much of the supernatant as possible, without disturbing the settled tissue pieces.
37. Transfer the supernatant into a 15 mL tube containing 3 mL of cell collection buffer. Keep at 4°C.
38. Add 1 mL of fresh enzyme mix to the Eppendorf with the remaining tissue pieces and repeat steps 33–37, combining the supernatant with the previously collected supernatant.

**CRITICAL:** Following these incubation and collection steps, the tissue pieces should be almost completely digested, with only residual white connective tissue remaining (which will be manually processed in step 39). If this is not the case, add 1 mL of fresh enzyme mix and repeat steps 33–37 for a third incubation.

39. Filter the supernatant through a 100 μm cell strainer, using the end of a syringe plunger to mechanically dissociate any remaining tissue pieces.
40. Rinse the cell strainer with cell collection buffer.
41. Centrifuge the collected cells at 400 × *g.*
42. Discard the supernatant and resuspend the cells to count, for example using Trypan blue exclusion and a hemocytometer.
  ***Note:*** An average yield of approximately 1.5 × 10^6^ cells is expected per tissue slice, although this will vary with the size of the lymph node and thereby size of the cross-section (expected range 0.5–4 × 10^6^ cells). Viability should be >70% (Figure 9).

43. Proceed with downstream applications.
  ***Optional:*** Human lymph node tissue slices often contain red blood cell contamination, which can be identified by staining with CD235a and sorted out by fluorescence-activated cell sorting if likely to interfere with downstream analyses (Figure 9).

## Expected outcomes

The size and shape of human lymph nodes vary (Figure 1), which in turn influences: i) The slicing precision, i.e., the ability to generate consecutive full cross-sections, ii) The number of slices generated per lymph node, and iii) The cell yield following digestion. A human cystic lymph node yields approximately 10–20 good quality tissue cross-sections. Each digested slice yields several million leukocytes (average 1.5 × 10^6^ cells, expected range 0.5–4 × 10^6^ cells) after digestion, of which the majority are B and T lymphocytes, but also includes rarer innate cells in addition to non-hematopoietic stromal cells (Figure 9). These cells are retained in tissue slices during short-term culture (<20 h), however cells also egress into the media and may be analyzed separately as the tissue-free portion (Figure 6). The lymph node tissue architecture, such as B cell follicles, T cell-rich paracortical regions and lymphatics, are retained within the tissue slices over short-term culture submerged in culture media (Figure 8). Slices from the same donor may be cultured as control and treatment conditions, allowing observation of donor-to-donor variation, but should be performed in replicate to control for slice-to-slice variation (Figure 7). Control slices of human lymph nodes show basal cytokine secretion into the supernatant (Figure 7), indicating a potential response to precision cutting and demonstrating that even uninflamed lymph nodes are active – likely reflecting the non-sterile environment of the human body.

## Limitations

Given the variability in shape, size and tissue consistency of human lymph nodes, precision and ease of cutting also varies between donors. This protocol provides optimized steps for a majority of lymph nodes but may require further adjustments in certain situations. Precision slicing will unavoidably result in the release of danger associated molecular patterns (DAMPs) which may prime or activate the more sensitive cell types, for example innate lymphoid cells. A large number of cells are lost during cutting and can be seen being released into the buffer tray. Cells lost from the tissue during culture can be collected and analyzed separately by collecting the culture supernatant. In particular, a loss of macrophages during culture has been observed. Slice-to-slice variation in response to stimuli is also observed, likely reflecting the capture of different cell types and microanatomical features. This can be controlled for, as far as possible, by the pooling of technical replicates. Under these conditions, slices remain viable over short-term culture (<20 h), while further optimization of cell media conditions, such as by perfusion^9^ or hydrogel-embedding,^4^ is likely required for longer term culture.

## Troubleshooting

### Problem 1: Lymph node falls out of the agarose block during cutting

Insufficient embedding of the lymph node in step 6 can cause it to fall out of the agarose during cutting. Re-embedding is possible, but it should be noted that the interior of the lymph node will then have been exposed to agarose, as opposed to embedding of full lymph nodes in which only the capsule is exposed, with potential impacts on cell viability and function.

### Potential solutions

A brief digitonin wash of samples before embedding removes excess lipids from the sample surface which can prevent complete embedding.^6^ Alternatively, for larger specimens in particular, the tissue can be superglued to the specimen tube base before adding the agarose to hold the lymph node in place. It should be noted, however, that this will prevent cutting of the full length of the tissue, as the blade cannot cut fully to the specimen tube base.

### Problem 2: Poor-quality slices

Variation in the type of lymph node (species, anatomical location, disease type, tissue density) may require further optimization of embedding and, particularly, cutting settings in steps 1–20 to produce consistently good quality slices. The blade may not cut fully through the tissue, resulting in uneven slices.

### Potential solutions

• The density of the agarose should be matched as closely as possible to the density of the tissue for successful cutting, thus increasing the agarose concentration may improve the cutting precision. Speed and oscillation settings can also be adjusted, with a decrease in cutting speed and increase in oscillation often improving the slice evenness.
• Regularly changing the blade will also improve cutting, as well as ensuring the blade is flat and straight (Figure 4). The use of a ceramic blade, instead of a standard stainless-steel blade, may be preferable for a given tissue type. Residual connective tissue may be manually trimmed with scissors from within the buffer tray, to prevent blade snagging.
• Smaller samples are easier to cut, and therefore tissue samples may also be cut into smaller sections prior to embedding, although this will result in the selection of tissue microanatomical features rather than providing an organ cross-section. Full cross-sections should be possible with human lymph nodes of approximately ≤1 cm in length.

### Problem 3: Tissue slice falls out of agarose during cutting

The tissue slice may not be retained in the agarose during cutting and collection in steps 20–21 and may fall out into the buffer tray.

### Potential solutions

Depending on the downstream application this may not necessarily be a problem, negating the need for steps 22–24. Instead, slices can be directly collected from the buffer tray and placed into culture media. Care should be taken while transferring the tissue, using flat headed forceps and/or a tissue brush, as the tissue in this case must be touched directly rather than transferred via the agarose. Improving embedding of the whole tissue within the agarose block (Troubleshooting 1) should also improve retention of tissue slices during cutting, if this is required.

### Problem 4: Loss of cell types during culture

Depending on the culture conditions, immune stimulant and culture duration, specific cell types may become more susceptible to cell death and loss during downstream analyses, in step 28.

### Potential solutions

Careful optimization of culture media composition, including potential oxygen perfusion^9^ or culture in hydrogels,^4^ may better sustain cell viability, especially over longer culture periods. Titration of immune stimulants is essential to determine the optimal concentrations for balancing immune activation with activation-induced cell death. Slices may be pooled within conditions to enrich for certain cell types. Alternatively, specific cell types (e.g., T and B cells) can be depleted to allow enrichment of rarer cell types.^1^ Culturing of slices on permeable inserts at the liquid-air interface may also reduce the level of cell egress without loss of tissue viability.

### Problem 5: Formalin-fixed tissue slices do not remain flat during paraffin embedding

Given the small size of the tissue slices, and their relative flexibility even after fixation, it can be difficult to retain the slice in the correct orientation and ensure it is flat during the paraffin embedding process, in step 28b, resulting in uneven tissue representation during further sectioning and immunohistochemical analysis.

### Potential solutions

**Figure 10:**
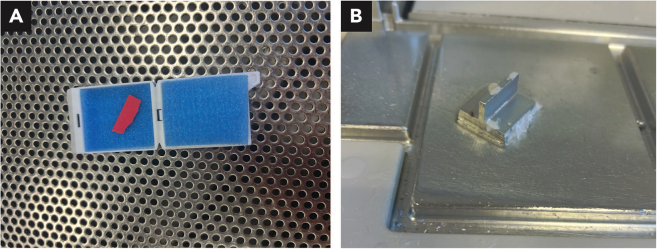
Paraffin Embedding (A) Embedding cassette with two biopsy sponges used to flatten tissue slice, represented by paper slip, during dehydration, clearing and paraffin infiltration. (B) Metallic press used to flatten tissue to the bottom of the mold during paraffin embedding.

Use a tissue brush to flatten and orientate the tissue slice between two biopsy sponges within the embedding cassette for dehydration, clearing and paraffin infiltration. During the subsequent paraffin embedding, press the flattened tissue to the bottom of the mold, using the metallic press (Figure 10).

## Resource availability

### Lead contact

Further information and requests for resources and reagents should be directed to and will be fulfilled by the lead contact, Joannah R. Fergusson (joannah.fergusson@kennedy.ox.ac.uk).

### Technical contact

Technical questions on executing this protocol should be directed to and will be answered by the technical contact, Joannah R. Fergusson (joannah.fergusson@kennedy.ox.ac.uk).

### Materials availability

This study did not generate new unique reagents.

### Data and code availability

The published article includes links to all datasets generated during this study. Any further data reported will be shared by the lead contact upon request.

## Acknowledgments

This work was funded by 10.13039/100014989Chan Zuckerberg Initiative grant 2020-217289 (J.R.F., M.C., C.A.D., and A.M.), 10.13039/501100000265UK Medical Research Council MR/Y004450/1 (J.R.F. and M.C.), 10.13039/100010269Wellcome Trust and Royal Society 204290/Z/16/Z (C.A.D.), John Fell Fund grant (A.M.), 10.13039/100000865Bill and Melinda Gates Foundation “Adjuvants for Global Health” grant INV001759 (A.M.), 10.13039/501100000265UK Medical Research Council MR/T030410/1 (C.A.D.), 10.13039/501100000833Rosetrees Trust R35579/AA002/M85-F2 (C.A.D.), 10.13039/100005565Janssen Biotech, Inc. Cartography Consortium (C.A.D.), 10.13039/100014461NIHR Biomedical Research Centre, Inflammation Across Tissues Theme (J.R.F. and C.A.D.), and 10.13039/100016580Kennedy Trust for Rheumatology Research.

We thank Anna Schepers for technical discussions, the provision of photographs and movies, and proofreading of the manuscript and Panyu Fei for further proofreading. We thank Rebecca Pompano and her lab members for foundational work that informed the development of this protocol. We also thank the Kennedy Institute facility staff, including the Flow Facility, Single Cell Facility, Digital Pathology Omics Core, Oxford Zeiss Centre of Excellence, and the Histology Facility, for technical assistance. Sample collection was supported by NIHR Biomedical Research Centre, Oxford and we thank the Oxford Translational Gastroenterology Unit Investigators for their help. The views expressed are those of the authors and not necessarily those of the NHS, the NIHR or the Department of Health. Adjuvant LMQ used in this study was provided by the Vaccine Formulation Institute, Switzerland. The graphical abstract was created using Biorender.com.

## Author contributions

J.R.F. developed and drafted the protocol, which was reviewed and edited by A.M. The project was supervised by A.M., M.C., and C.A.D.

## Declaration of interests

The authors declare no competing interests.

## Supplemental information

Methods video S1. Agarose trimming prior to tissue slicing with the Compresstome® VF-510-0Z, related to steps 16 and 17

Methods video S2. Precision cutting of live lymph node with the Compresstome® VF-510-0Z, related to step 20

Methods video S3. Accelerated video of consecutive slicing and collection of live lymph node slices with the Compresstome® VF-510-0Z, related to step 21

## References

[1] Fergusson J.R., Siu J.H.Y., Gupta N., Jenkins E., Nee E., Reinke S., Ströbel T., Bhalla A., Kandage S.M., Courant T. (2025). Ex vivo model of functioning human lymph node reveals role for innate lymphocytes and stroma in response to vaccine adjuvant. Cell Rep..

[2] Belanger M.C., Ball A.G., Catterton M.A., Kinman A.W.L., Anbaei P., Groff B.D., Melchor S.J., Lukens J.R., Ross A.E., Pompano R.R. (2021). Acute Lymph Node Slices Are a Functional Model System to Study Immunity Ex Vivo. ACS Pharmacol. Transl. Sci..

[3] Salmon H., Rivas-Caicedo A., Asperti-Boursin F., Lebugle C., Bourdoncle P., Donnadieu E. (2011). Ex vivo imaging of T cells in murine lymph node slices with widefield and confocal microscopes. J. Vis. Exp..

[4] Fernando K., Quah H.S., Suteja L., James A., Kuthubudeen F.F., Wu K.Z., Adine C., Bhuvaneswari H., Senthilkumar M., Selvarajan S. (2025). Extended human lymph node explants for evaluation of adaptive immunity. Trends Biotechnol..

[5] O'Donnell J.S., Isaacs A., Jakob V., Lebas C., Barnes J.B., Reading P.C., Young P.R., Watterson D., Dubois P.M., Collin N., Chappell K.J. (2022). Characterization and comparison of novel adjuvants for a prefusion clamped MERS vaccine. Front. Immunol..

[6] Ball A.G., Belanger M.C., Pompano R.R. (2021). Detergent wash improves vaccinated lymph node handling ex vivo. J. Immunol. Methods.

[7] Donovan M.L., Jhaveri N., Ma N., Cheikh B.B., DeRosa J., Mihani R., Berrell N., Suen J.Y., Monkman J., Fraser J.F., Kulasinghe A. (2024). Protocol for high-plex, whole-slide imaging of human formalin-fixed paraffin-embedded tissue using PhenoCycler-Fusion. STAR Protoc..

[8] Maiques O., Sanz-Moreno V. (2022). Multiplex chromogenic immunohistochemistry to stain and analyze paraffin tissue sections from the mouse or human. STAR Protoc..

[9] Anbaei P., Stevens M.G., Ball A.G., Bullock T.N.J., Pompano R.R. (2024). Spatially resolved quantification of oxygen consumption rate in ex vivo lymph node slices. Analyst.

